# Changes in methylation associated with development of metabolic syndrome in testicular cancer patients treated with cisplatin chemotherapy

**DOI:** 10.1038/s41598-025-22918-y

**Published:** 2025-11-11

**Authors:** Marcin W. Wojewodzic, Ellen L.D. Volders, Coby Meijer, Tom Grotmol, Daan J. Touw, Sjoukje Lubberts, Trine B. Haugen, Jourik A. Gietema, Trine B. Rounge

**Affiliations:** 1 Department of Research, Cancer Registry of Norway, Norwegian Institute of Public Health, Oslo, Norway; 2 Division for Climate and Environmental Health, Department of Chemical Toxicology, Norwegian Institute of Public Health, Oslo, Norway; 3 Medical Oncology, University of Groningen, Groningen, The Netherlands; 4 Pharmaceutics and Pharmacology, University Medical Centre Groningen, University of Groningen, Groningen, The Netherlands; 5 Department of Life Sciences and Health, Faculty of Health Sciences, Oslo Metropolitan University, Oslo, Norway; 6 Department of Pharmacy, Faculty of Mathematics and Natural Sciences, University of Oslo, Oslo, Norway

**Keywords:** Testicular germ cell tumour, Metabolic syndrome, Cisplatin, Survivorship, Epigenomics, Methylation, Germ cell tumours, Testicular cancer, Urological cancer, Cancer, Metabolic disorders, Cancer, Systems biology, Biomarkers, Diseases, Health care, Medical research, Molecular medicine, Oncology, Risk factors

## Abstract

Patients with metastatic testicular cancer (TC) treated with cisplatin-based chemotherapy (CBCT) are prone to develop metabolic syndrome (MetS). In an epigenome wide association study in patients with TC we have shown that DNA methylation was associated to CBCT. The aim of the study was to investigate whether there was a change in DNA methylation after treatment with CBCT, and if methylation status is related to the presence or development of MetS. In a prospective cohort of 67 TC patients who received CBCT, we assessed whole blood global methylation long interspersed nuclear element-1 (LINE-1) and DNA methylation at selected cytosine-phosphate-guanine dinucleotide (CpG) sites associated with MetS using targeted sequencing. Measured samples were taken before, one month after, and one year after CBCT. Development of MetS was assessed before and up to five years after CBCT. Serum platinum levels were measured to assess platinum exposure (PtAUC) within the first year after the start of CBCT. Data were used for paired comparisons, comparisons between groups, statistical modeling to account for covariates, and machine learning approaches to predict occurrence of MetS five years after diagnosis. Global methylation did not change during the first year after the start of CBCT (median 73.7% (25th -75th percentile 72.0–75.2) vs. 73.9% (72.5–75.1)). Seven out of the 16 other selected CpGs decreased. Patients with MetS before start of CBCT (*N* = 18) showed a larger decrease than patients without MetS (*N* = 49) for AC090023 (-11.6% vs. -8.2%, *p* = 0.008), NCAM2 (-6.5% vs. -1.6%, *p* = 0.030), and TOM1L2 (-5.8% vs. -2.6%, *p* = 0.003). The 15 patients who did not have MetS prior to CBCT, but developed MetS within five years after treatment showed an increase in global methylation (1.5% vs. − 0.6%, *p* = 0.008) after one year, and a decrease in TOM1L2 methylation (-4.0% vs. -1.6%, *p* = 0.015) when compared to patients who did not have prior to CBCT nor developed MetS (*N* = 34). There was no difference in PtAUC during the first year after the start of CBCT between the latter groups (70.0 vs. 67.9 days*mg/L, *p* = 0.206). Changes in global DNA methylation and TOM1L2 one year after the start of CBCT are associated with a higher risk for newly developing MetS within five years. This may indicate potential for tailored advice, based on epigenetic status, to patients treated with chemotherapy for TC to prevent development of MetS.

**Supplementary Information:**

The online version contains supplementary material available at 10.1038/s41598-025-22918-y.

## Introduction

Since the late 1970s testicular cancer (TC) patients with disseminated disease have been treated with cisplatin-based chemotherapy (CBCT)^1^. The introduction of CBCT has led to a very good prognosis, resulting in an increase in the number of survivors of TC, with a 5-year overall survival rate of over 85–95% in Northern European countries^2^. However, during CBCT bone marrow suppression, nephrotoxicity, ototoxicity, neurotoxicity, infertility and cardiovascular events may occur^2^, these are also well-recognized late effects of cancer treatment. In addition, late effects of CBCT can develop over time, comprising second primary malignancies, overweight, hypogonadism, metabolic syndrome (MetS), and cardiovascular diseases, resulting in lower quality of life on the long-term^3,4^. In addition, late effects may lead to premature death^5^.

Development of these late effects of CBCT could be a consequence of changes in methylation, initiated during, shortly after or long after cytotoxic treatment. Other factors, such as smoking, physical inactivity, and overweight, may also contribute to the late effects of CBCT, indicating that these outcomes are not solely attributable to methylation changes^6,7^. A Norwegian study among TC survivors (*N* = 279) showed that patients treated with CBCT 16 years earlier had changes in methylation at 35 different CpG sites, when compared to patients treated without CBCT and adjusted for pivotal covariates, such as age, smoking and blood cell composition. These changes in methylation sites were close to genes that are related to insulin metabolism and body mass. A direct link between CBCT or MetS and methylation in this study was however not found. The role of DNA methylation in the complex relationship between TC, CBCT and MetS is still largely unexplored, and the impact of time and dose of CBCT on methylation and risk for developing MetS needs to be investigated. The Dutch prospective cohort of TC survivors containing follow-up data on MetS status from prior diagnosis, and up to five years after CBCT have samples and platinum measurements ideal for exploring these questions^8^.

Overall, our aim was to validate and explore observed changes in methylation in TC patients treated with CBCT and to further investigate the (sub-)acute effects of CBCT.

**Fig. 1:**
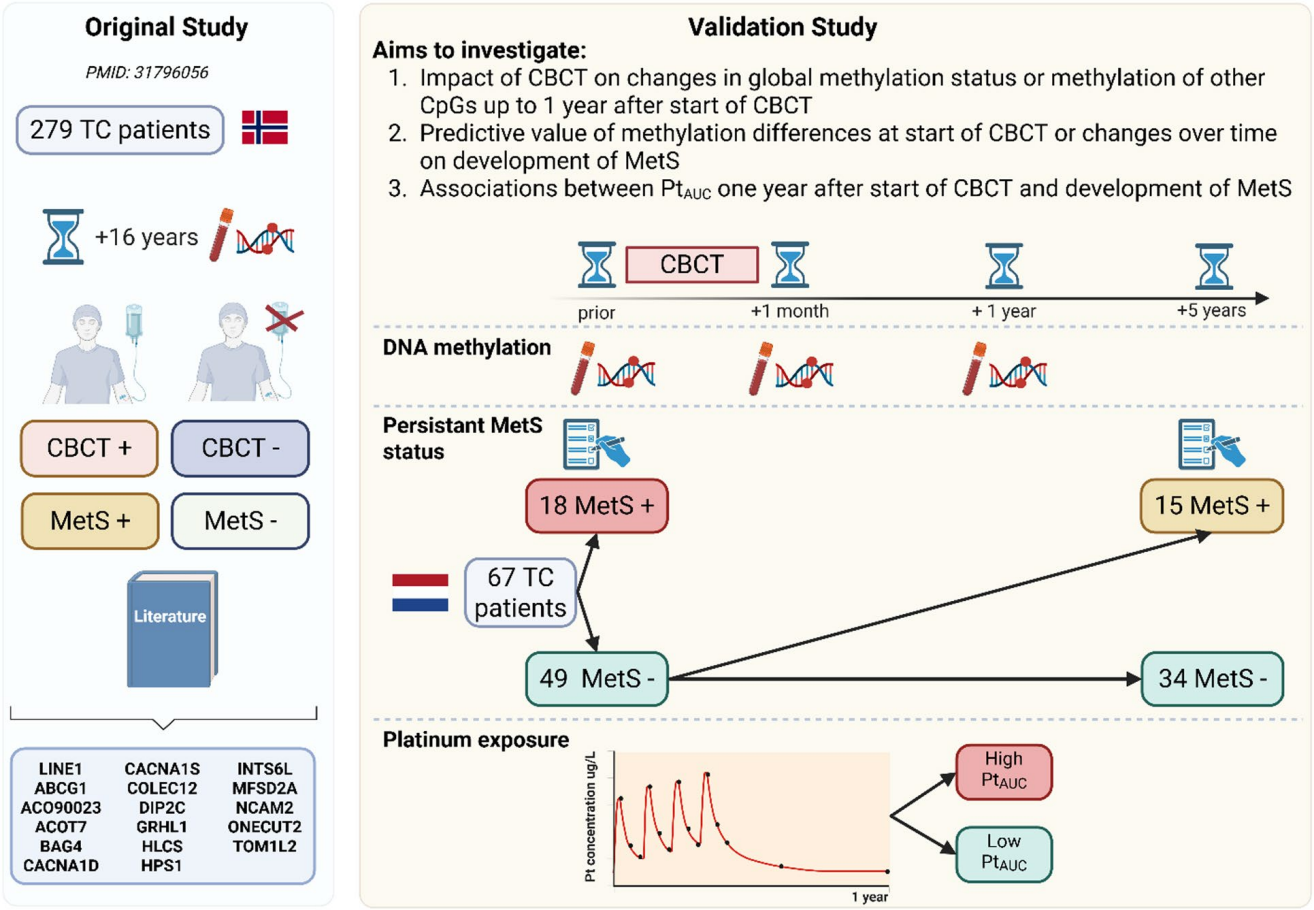
Overview of the study design. Left panel: The original discovery study involved Norwegian TC patients that resulted in CpGs list candidates associated with the effects of CBCT and MetS, 16 years after CBCT. Additionally, literature studies were conducted to support the selection of candidates for replication studies. Right panel: In this prospective study on TC survivors from the Netherlands, DNA methylation levels were measured before start of CBCT, 1 month after and 1 year after CBCT. Information on MetS was available prior to treatment and up to 5 years after the treatment. Pt_AUC_ was modeled by measurements of platinum levels within the first year after start of CBCT. The figure was created using BioRender.com. CBCT = cisplatin-based chemotherapy; MetS = metabolic syndrome; Pt_AUC_ = exposure to platinum measured as area under the curve (AUC); TC = testicular cancer. Definition of persistent MetS status: once a patient is diagnosed with MetS, there is no possibility to escape from this.

Specifically, this study aimed to investigate (1) whether there was a change in global methylation status or methylation of selected CpGs after treatment with CBCT (2) if methylation status or changes in methylation over time could be related to the presence or development of MetS, and (3) if differences could be associated with exposure to platinum (PtAUC). An overview of the study design is shown in Fig. 1.

## Results

Sixty-seven patients were included in this analysis, of which the basic characteristics can be found in Additional file 1 in the supplementary Table 1. More base characteristics can be found in the original study by Lubberts et al.^8^.

An overview of global methylation at long interspersed nuclear element-1 (LINE-1) and methylation of other selected MetS-related cytosine-phosphate-guanine dinucleotides (CpGs) before CBCT, one month after CBCT and one year after start of CBCT, and the number of patients in which paired comparisons between these time points were possible, can be found in Additional file 1 in the supplementary Table 2.

### Overall changes in methylation after cisplatin-based chemotherapy (CBCT)

**Fig. 2:**
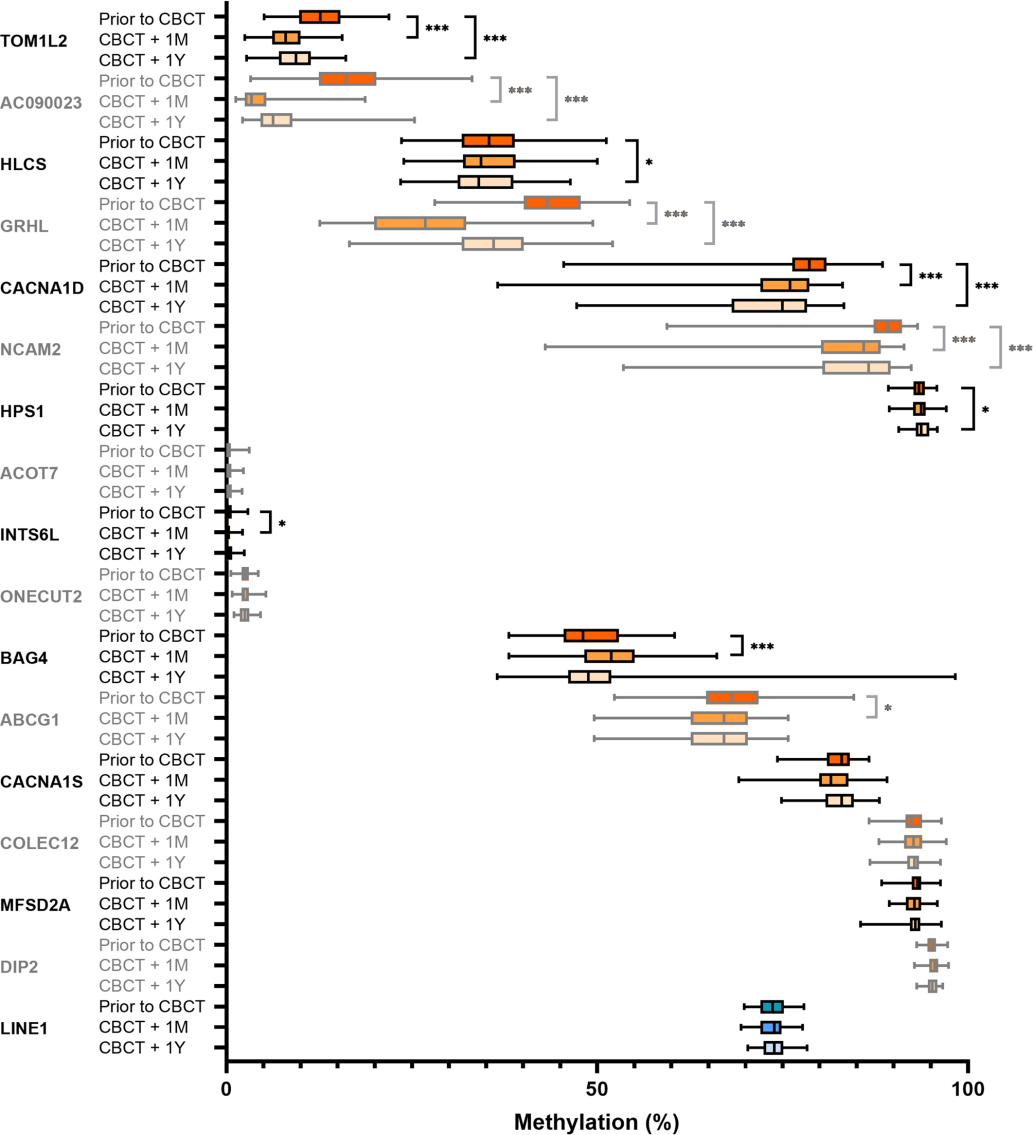
Box plots of methylation levels of targeted CpGs and LINE-1 (global methylation) at 3 different time points: prior to CBCT, one month after CBCT, and one year after start of CBCT. The box plots are sorted by methylation status. Top 7 CpGs were significant for difference in methylation between prior to CBCT and one year after start of CBCT. Significance marked as: ***= *p* ≤ 0.001, *= *p* ≤ 0.05. CBCT = cisplatin-based chemotherapy; +1 M = one month after CBCT; + 1Y = one year after start of CBCT.

Overall, global methylation (LINE-1) did not change one year after the start of CBCT (median 73.7% vs. 73.9%) compared with prior CBCT (Fig. 2).

Seven out of sixteen selected CpGs changed significantly one year after the start of CBCT (Fig. 2, Additional file 1 in supplementary Table 2). A significant decrease in methylation of the CpG sites was found for TOM1L2 (12.7% vs. 9.4%, p = < 0.001), AC090023 (median 16.2% vs. 6.3%, p = < 0.001), HLCS (35.4% vs. 34.0%, *p* = 0.048), GRHL1 (43.3% vs. 36.0%, p = < 0.001), CACNA1D (78.6 vs. 75%, p = < 0.001), and NCAM2 (89.2% vs. 86.6%, p = < 0.001), and a borderline significant increase in methylation was found for HPS1 (93.4% vs. 93.7%, *p* = 0.052).

Of the seven CpGs in which methylation had changed one year after chemotherapy, methylation of five was changed already one month after chemotherapy (Fig. 2, Additional file 1 in supplementary Table 2). These were AC090023, CACNA1D, GRHL1, NCAM2, and TOM1L2. We did not adjust for multiple comparisons given the low number of cases, which leave us with a larger type 1 error. After Bonferroni adjustment the p-value significance found in HLCS, HPS, INTS6 and ABCG1 were below the threshold.

### Changes in methylation and metabolic syndrome prior to start of CBCT

Out of 67 patients, 18 had already MetS prior to CBCT. Of the 49 patients without the presence of MetS prior to start of CBCT 15 were newly diagnosed with MetS within five years after CBCT by using the persistent MetS status variable (once MetS is diagnosed, there is no possibility to escape from this) (Fig. 1, Additional file 1 in supplementary Table 1).

**Fig. 3:**
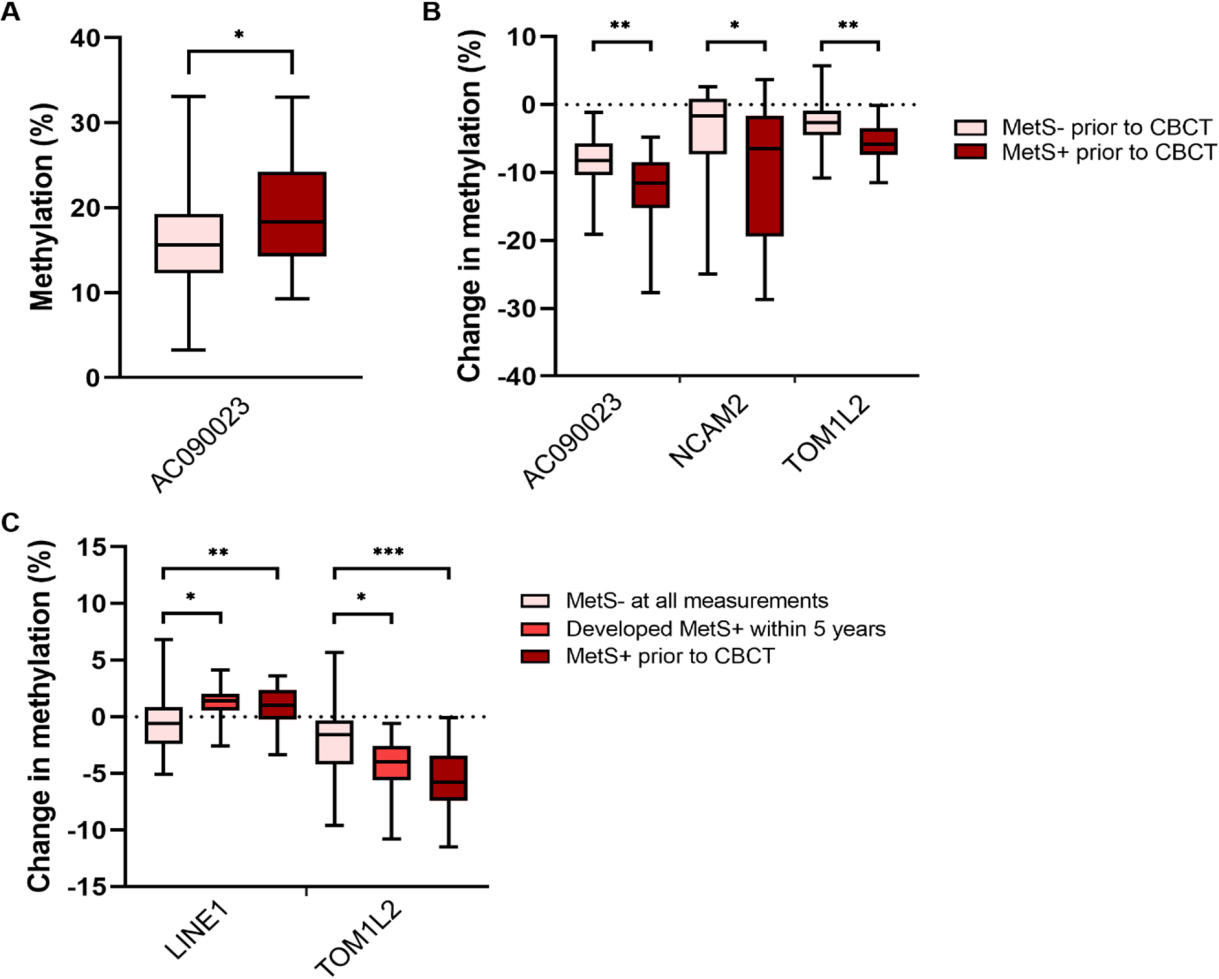
(**A**) Methylation of AC090023 in TC patients with or without MetS before the start of CBCT. (**B**) Changes in methylation of AC090023, NCAM2 and TOM1L2 one year after CBCT in TC patients with or without MetS before start of CBCT. (**C**) Changes in global methylation (LINE-1) and methylation of TOM1L2 one year after CBCT in TC patients who had MetS prior to CBCT, developed MetS within five years, or never developed MetS. * = *p* ≤ 0.05; ** = *p* ≤ 0.01; *** = *p* ≤ 0.001. CBCT = cisplatin-based chemotherapy; MetS = metabolic syndrome; TC = testicular cancer.

Patients who already had MetS prior to CBCT (*N* = 18) showed higher methylation levels in AC090023 (median 18.4% (25th-75th percentile 14.3–24.2) vs. 15.7% (12.3–19.3), *p* = 0.040) prior to CBCT, compared to patients who did not have MetS prior to CBCT (*N* = 49) (Fig. 3A). Global methylation (LINE-1) and other selected CpGs were not found to be different between these two groups. There were no differences found in age at diagnosis (33 years (27–41) vs. 30 years (25–35), *p* = 0.057) or leucocyte count (6.2 * 10^9^/L (5.2–7.8) vs. 6.2* 10^9^/L (5.3–7.2), *p* = 0.640)) before CBCT between patients that had MetS, and patients who did not have MetS.

Furthermore, these 18 patients with MetS prior to CBCT showed a larger decrease in methylation of AC090023, NCAM2 and TOM1L2 one year after CBCT, compared to patients who did not have MetS before this treatment (Fig. 3B); (-11.6% (-15.3 – -8.5) vs. -8.2% (-10.4 – -5.7), *p* = 0.007), (-6.5% (-19.4 – -1.6) vs. -1.6% (-7.3 – 0.9), *p* = 0.048) and (-5.8% (-7.4 – -3.5) vs. -2.6 (-4.5 – − 0.9), *p* = 0.004), respectively.

There were no differences between patients with MetS at start of CBCT and patients without MetS, in change in leucocyte count one year after the start of CBCT (-0.60 (-1.68 – 0.78) * 10^9^/L vs. − 0.90 (-1.75 – − 0.15)* 10^9^/L, *p* = 0.412) or platinum exposure (PtAUC) (66.4 (63.3–77.3) vs. 68.6 (65.2–82.7) days *mg/L, *p* = 0.141).

### Changes in methylation one year after the start of CBCT and development of metabolic syndrome within five years after the start of CBCT

Using the persistent MetS status variable, there was a significant difference found between patients who developed MetS within five years after the start of chemotherapy (*N* = 15) and patients who did not develop MetS (*N* = 34) for changes in global methylation (LINE-1) (median 1.5% (25th-75th percentile 0.6–2.0) vs. − 0.6% (-2.4 – 0.9), *p* = 0.034) and TOM1L2 (-4.0% (-5.6 – -2.6) vs. -1.6% (-4.2 – − 0.3), *p* = 0.022) (Fig. 3C) one year after CBCT. To confirm the statistical robustness of differences found in changes of methylation levels between groups for LINE-1 we performed Monte Carlo Simulation (further details can be found in Supplementary Fig. 2).

There were no differences found for changes in leucocyte count, or PtAUC during the first year after the start of CBCT between these groups (-0.90 * 10^9^/L (-2.10 – − 0.20) vs. − 0.90 * 10^9^/L (-1.55 – − 0.08), *p* = 0.670; 70.0 days *mg/L (66.1–88.2) vs. 67.9 days *mg/L (64.8–78.6) days *mg/L, *p* = 0.206).

In patients that had developed MetS within the first year after CBCT (*N* = 10), significantly higher PtAUC was found compared to patients without MetS (*N* = 39) (79.8 days *mg/L (68.3–96.7) vs. 67.7 days *mg/L (64.8–75.8), *p* = 0.049) (Additional file 1 in supplementary Tables 2, and supplementary Fig. 1).

### Changes in TOM1L2 after CBCT and development of metabolic syndrome

Patients who had the largest decrease in TOM1L2 (0-25th percentile) one year after the start of CBCT, more often had MetS before chemotherapy (9 out of 17, 53%) or newly developed MetS within 5 years after chemotherapy (3 out of 14, 21%), than patients without development of MetS after 5 years (3 out of 31, 10%) (*p* = 0.004).

### Linear modeling of development of metabolic syndrome by changes in CpGs

After adjusting for age at diagnosis, change in leucocyte count (standard laboratory technique; expressed as number x 10^9^/L) one year after start of CBCT (leukocyte count one year after start – leucocytes count before chemotherapy), and PtAUC, we found associations between MetS prior to CBCT or developed within 5 years, and the change in global methylation (LINE-1), AC090023, NCAM2 and TOM1L2 (Table 1). PtAUC was not a significant predictor in these four models.

**Table 1:** Ordinary least square (OLS) regression results with main terms used in the model and p-values for individual methylation changes one year after the start of CBCT compared to prior to CBCT.

	Intercept	MetS + prior to CBCT	MetS + developed within 5 years	Age	Pt_AUC_	Leucocyte change
Δ LINE-1	1.97 *p* = 0.312	1.57 p = 0.032*	1.89 p = 0.017*	-0.076 *p* = 0.080	1.6*10^− 10^ *p* = 0.994	0.24 *p* = 0.186
Δ AC090023	-6.33 *p* = 0.101	-6.29 p = 0.001**	-2.37 *p* = 0.123	0.10 *p* = 0.223	-5.7*10^2^ *p* = 0.195	0.37 *p* = 0.277
Δ CACNA1D	4.38 *p* = 0.460	-4.04 *p* = 0.061	1.10 *p* = 0.468	0.020 *p* = 0.875	-1.00*10^1^ *p* = 0.110	1.64 p = 0.004**
Δ NCAM2	11.17 *p* = 0.084	-5.75 p = 0.014*	-1.32 *p* = 0.604	-0.21 *p* = 0.141	-9.8*10^2^ *p* = 0.184	0.98 *p* = 0.095
Δ TOM1L2	1.35 *p* = 0.602	-3.39 p = 0.001**	-1.41 *p* = 0.176	-0.002 *p* = 0.974	-4.69*10^2^ *p* = 0.120	0.32 *p* = 0.174

MetS = Metabolic syndrome; Pt_AUC_ = Platinum exposure. Significance *p* ≤ 0.05 is marked with bold text and *, significance *p* ≤ 0.005 is marked with bold text and **.

After removal of the 18 patients who already had MetS prior to CBCT, changes in methylation of LINE-1 were significantly different for patients that developed MetS within 5 years (Table 2). Changes in other CpGs were not significantly associated. However, we found Pt_AUC_ and leucocyte change were significant predictors for change in methylation of TOM1L2.

**Table 2:** Ordinary least square (OLS) regression results with main terms used in the model and p-values for individual methylation change between prior to CBCT and one year after CBCT, excluding MetS patients prior to CBCT.

	Intercept	MetS + developed within 5 years	Age	Pt_AUC_	Leucocyte change
Δ LINE-1	1.75 *p* = 0.186	1.85 p = 0.033*	-0.062 *p* = 0.295	-1.58*10^3^ *p* = 0.953	0.30 *p* = 0.263
Δ AC090023	-7.06 *p* = 0.073	-2.30 *p* = 0.090	0.017 *p* = 0.854	-1.05*10^2^ *p* = 0.807	0.57 *p* = 0.197
Δ CACNA1D	6.39 *p* = 0.258	-1.23 *p* = 0.535	-0.019 *p* = 0.891	6.25*10^2^ *p* = 0.090	2.70 p = 0.001**
Δ NCAM2	13.13 p = 0.023*	-1.71 *p* = 0.553	-0.24 *p* = 0.092	-0.1*10^− 6^ *p* = 0.083	1.11 *p* = 0.087
Δ TOM1L2	3.69 *p* = 0.197	-1.27 *p* = 0.210	-0.020 *p* = 0.775	-6.77*10^2^ p = 0.040*	0.67 p = 0.044*

LINE-1 and AC090023 are shown for transparency. Significance *p* ≤ 0.05 is marked with bold text and *. significance *p* ≤ 0.005 is marked with bold text and **.

### Predicting occurrence of development of metabolic syndrome within 5 years after CBCT using machine learning algorithms

**Fig. 4:**
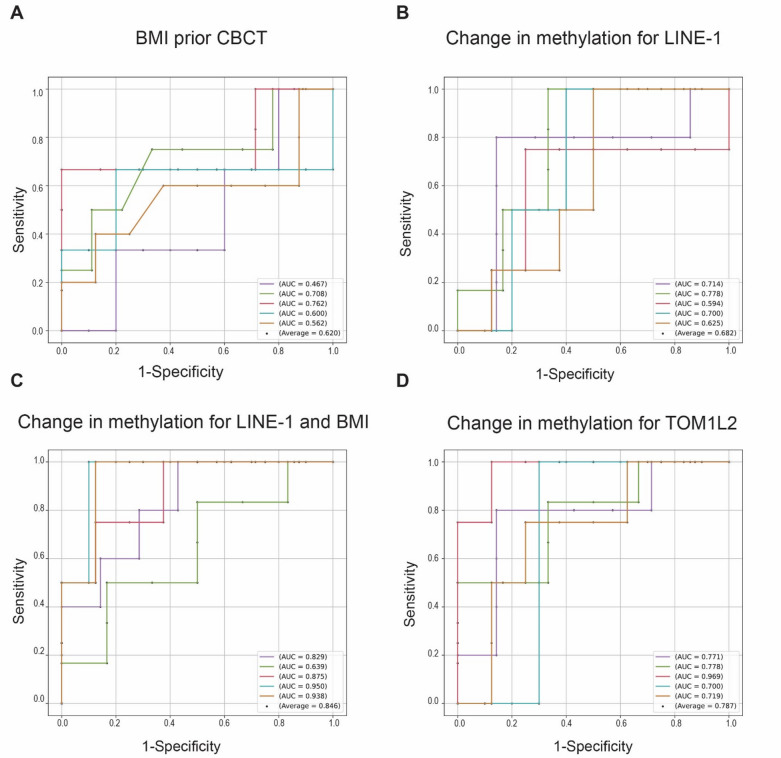
Receiver Operating Characteristic (ROC) curves illustrating the performance of four predictor sets for developing MetS within 5 years after CBCT. The following sets were used: BMI at the start of CBCT (**A**), change in LINE-1 methylation one year after CBCT (**B**), the combination of these two (**C**), and change in TOM1L2 one year after CBCT (**D**) in a 5-fold cross-validation framework. ROC curve generated for a respective predictor set, demonstrating their discriminative ability for predicting the development of MetS within five years after CBCT. Average AUC is also displayed. AUC = area under the curve; BMI = body mass index; CBCT = cisplatin-based chemotherapy; MetS = metabolic syndrome.

To further explore methylation markers that could help predict the development of MetS within 5 years after CBCT, machine learning with 5-fold cross-validation was utilized. First, we assessed the standalone predictive performance of Body Mass Index (BMI) at the start of CBCT (Fig. 4A), to uncover its moderate potential as a predictor of MetS developed 5 years after CBCT. BMI alone had an average AUC of 0.62. We evaluated the predictive power of the difference in LINE-1 methylation between one year after the start of CBCT and prior to CBCT (Fig. 4B) for the development of MetS within 5 years after CBCT. The prediction power of LINE-1 had an average of 0.68 in AUC. We further investigated the combined predictive capacity of BMI and LINE-1 methylation (Fig. 4C), which improved AUC to 0.85. Finally, AUC for TOM1L2 methylation had (Fig. 4D) 0.79 in AUC. When employing methylation data for the 5 most prominent CpGs (LINE-1, TOM1L2, AC090023, NCAM2, CACNA1D), we did not observe further improvement of the models (average AUC 0.73).

### Changes in TOM1L2 one year after CBCT stratified by amount of platinum exposure within the first year after CBCT and development of metabolic syndrome within five years

**Fig. 5:**
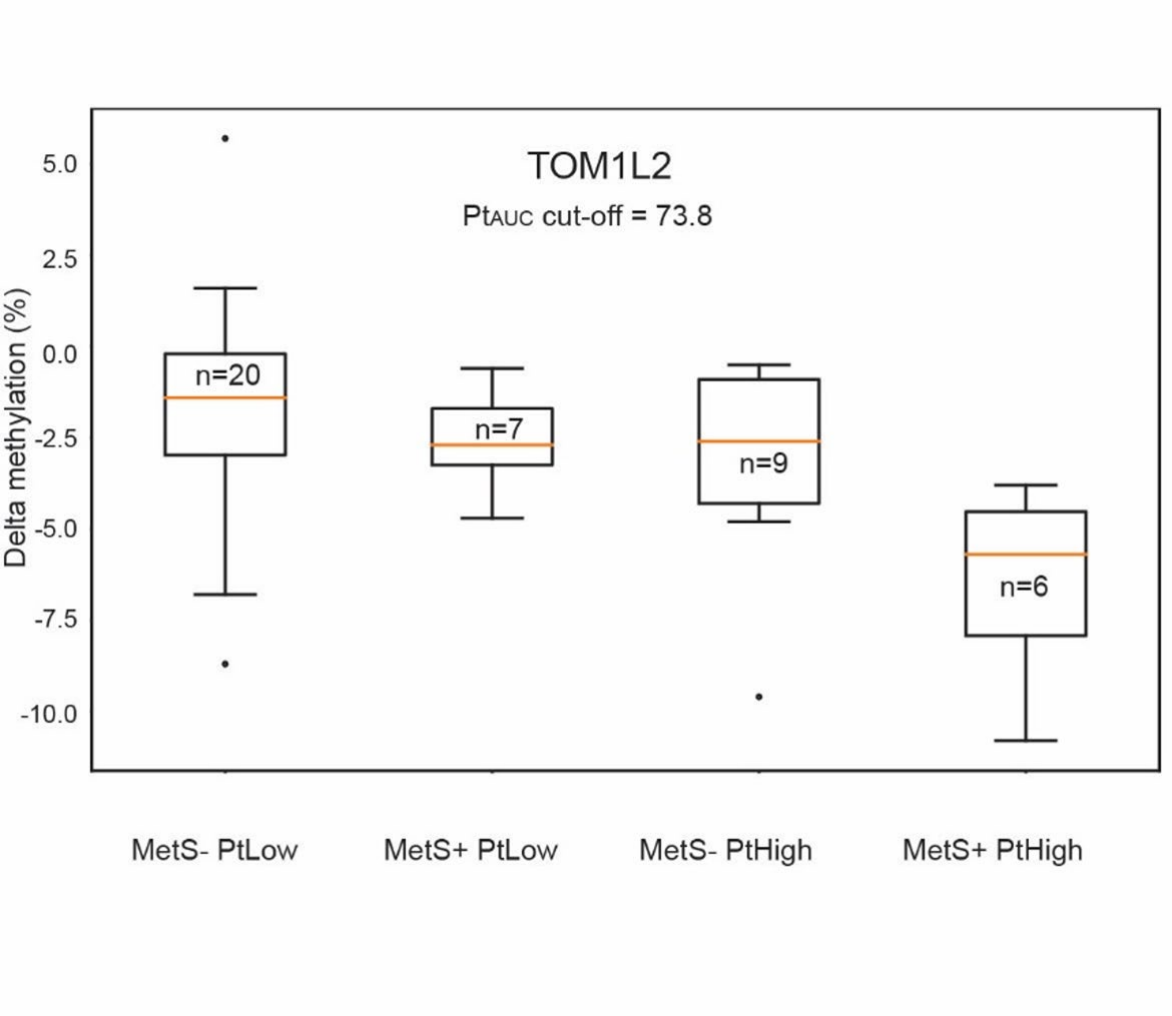
Changes in TOM1L2 methylation levels one year after CBCT stratified by Pt_AUC_ and development of MetS within 5 years after CBCT. CBCT = cisplatin-based chemotherapy; MetS = metabolic syndrome; Pt_AUC_ = exposure to platinum measured as area under the curve. PtHigh = group above the cut-off, PtLow = group below the cut-off.

To investigate if any changes in TOM1L2 could be associated with development of MetS and Pt_AUC_, we stratified patients into four groups. First, by using the persistent MetS status: patients that had developed MetS within 5 years after CBCT, and patients without MetS. Second, by using Pt_AUC_ during the first year after CBCT, distinguishing between high and low Pt_AUC_ using a cutoff mean AUC of 73.8 days *mg/L (Fig. 5). While the differences were not statistically significant (Fstat = 0.376), we noticed a trend that patients with the largest decrease in TOM1L2 methylation and highest Pt_AUC_ were more likely to develop MetS within 5 years after CBCT.

## Discussion

This validation study in a Dutch prospective cohort of TC survivors, showed that global methylation status does not change while AC090023, NCAM2 and TOM1L2 decreased the first year after CBCT. This decrease was larger in patients that already had MetS at the start of CBCT. Patients that developed MetS within five years after the start of chemotherapy showed increased global methylation after one year and a larger decrease in TOM1L2 methylation than patients that never developed MetS. Development of MetS within five years was not associated with higher exposure to platinum (Pt_AUC_) in the first year after the start of CBCT.

So far, research on methylation changes in TC has been focusing on their relationship to cisplatin sensitivity or resistance^9^, and disease prognosis^10–12^. Studies by Lobo et al. showed higher methylation of several genes in non-seminoma, in patients with highly differentiated tumors and higher tumor burden^13^, and in patients with platinum resistant disease^14^. However, it remains unclear if the CpGs that were significantly changed after CBCT in our study, match any of those found in these other studies.

We found an increase in LINE-1 methylation one year after the start of CBCT in patients that developed MetS within five years after chemotherapy. A review by Singh et al. reports on demethylation of LINE-1 elements in the presence of TC in tumor tissue, which may explain an increase in methylation when the cancer is in remission^15^. Furthermore, studies in individuals with obesity or cardiometabolic disease are still conflicting concerning changes in global methylation as described in a systematic review in the general population^16^. Three out of nine studies observed increases in LINE-1 methylation^17–19^, whereas four found decreases^20–23^, and two did not find any correlation^24,25^.

Our study focused primarily on changes in methylation one year after chemotherapy, but there were also methylation changes seen in several other CpGs already one month after completion of CBCT. These acute changes may be related to alteration in blood cell composition due to bone marrow suppression, since the sampling of blood has been performed only one month after completion of CBCT. A study performed by Reinius et al. showed large differences in methylation status within different blood cell types^26^. Another study by Sadahiro et al. showed blood cell composition changes shortly after major surgery^27^. Since we observed major changes in leucocyte count during CBCT, methylation status at that time point was not further analyzed to avoid artifacts. However, the CpGs that changed one month after CBCT were partly comparable to changes one year after CBCT. Whether this is a direct effect from CBCT or an indirect effect of not fully recovered blood cell composition shortly after treatment is unclear. For further analyses, changes one year after CBCT were used, because the patients are in a more stable state and without any manifestation of bone marrow suppression supported by clinical measurements. However, some patients had lower leucocyte count measured than before CBCT, which is why this possible confounder was added to our models. By that, we found that change in TOM1L2 was significantly associated with a change in leucocyte count.

In the original study by Bucher-Johannessen et al.^28^, TC survivors treated with CBCT 16 years ago showed differences in methylation in 35 CpG sites, compared to patients not treated with CBCT. In this validation, we were able to investigate the (sub-)acute influence of CBCT on changes in methylation and the development of MetS, by using Pt_AUC_ during the first year after start of treatment. We did not find a significantly different Pt_AUC_ in the patients that had developed MetS within 5 years after treatment, when testing univariately. However, patients that already had developed MetS within the first year after CBCT, did show higher Pt_AUC_, indicating a (sub-)acute effect of CBCT. Also, higher Pt_AUC_ was associated with a larger decrease in TOM1L2. The decrease in TOM1L2 was largest in patients that also developed MetS within 5 years.

To assess the predictive efficacy of available variables for the development of MetS within five years after CBCT, we tested multiple models by utilizing machine learning. Since BMI is a well-known predictor for the presence of MetS, we considered a possible autocorrelation between BMI at the start of CBCT and the development of metabolic syndrome within five years. BMI demonstrated a moderate AUC. Also, alterations in LINE-1 methylation status did not enhance AUC by itself. By combining these two, we were able to improve this predictability. This was also the case for change in TOM1L2 one year after CBCT. These novel findings indicate that there are patients without a high BMI that develop MetS, and that incorporation of methylation status of TOM1L2 or LINE-1 may contribute to identify patients at highest risk for developing MetS after CBCT. In these identified high-risk patients, intervention strategies to prevent the development of MetS can be investigated. Validating these results in an additional, independent cohort of patients treated with cisplatin containing chemotherapy would be valuable, as our small sample size carries a risk of overfitting.

The role of TOM1L2 in MetS remains uncertain, but emerging evidence suggests potential links with metabolic traits. Based on its known function in endosomal trafficking, TOM1L2 may influence glucose and lipid homeostasis by regulating the recycling of metabolic components such as insulin receptors or lipid transporters^29^. Additionally, genetic variants near the TOM1L2/SREBF1 locus have been associated with body composition traits, and the proximity to SREBF1 (a key regulator of lipid synthesis) suggests possible shared regulation of metabolic pathways^30^.

One of the strengths of this study, is that it is the first prospective longitudinal study in TC patients that has analyzed (sub-)acute changes in methylation status after CBCT and has been used for the predictive purposes of developing MetS. By using the persistent MetS status variable that reflects the development of MetS instead of its presence at a certain time point, we were able to see changes within patients and patient groups.

Due to the longitudinal approach employed in this study, which involved paired sampling, and considering the rarity of TC, the study population remained limited, which constitutes a notable constraint.

Additional investigations should extend the analyses of TOM1L2 protein measurements to a larger patient population, verifying the robustness of this association. Alternatively, the implementation of quantitative methylation assays for TOM1L2 on a broader group could be designed. Importantly, as we have successfully replicated the predictive power of TOM1L2 in our current study, comparing favourably to Boucheron et al. 2019, it suggests that the observed association remains consistent^28^.

## Conclusions

In this validation study we found that TC patients that were treated with CBCT show changes in several methylation sites, including AC090023, CACNA1D, GRHL1, HLCS, NCAM2, TOM1L2, HPS1. These changes are induced either by a direct effect of treatment with cisplatin, causing true differences in methylation status, or an indirect effect, such as changes in leucocyte count. Further exploration found that patients with large decreases in TOM1L2 methylation and high platinum exposure within the first year after start of CBCT, more often develop MetS. Measuring TOM1L2 may help to identify TC patients at the highest risk of developing MetS. Without confirmation in larger patient cohorts on protein level, the findings should be interpreted with caution. Finally, cost-benefit analyses should also be conducted to evaluate the clinical utility of these findings.

## Materials and methods

### Study participants

All methods were performed in accordance with the relevant guidelines and regulations. Participants were part of a prospective study on cardiovascular disease risk during the first year of CBCT treatment in TC patients. This study was performed in the University Medical Centre Groningen (UMCG) between 2008 and 2012 and approved by the Research Ethics Committee of the UMCG (Fig. 1). In addition, this study was also approved by Regional Ethical Committee (REC) south east D in Norway, reference 2015/1332. All participants have given informed consent. A detailed description of the study design is given elsewhere^8^.

In short, patients were found eligible when aged between 18 and 50 years, had to start with 3 or 4 cycles of first-line (B)EP (bleomycin, etoposide, cisplatin) chemotherapy and had no history of cardiovascular events. Study visits with blood sample collections were performed at the time of diagnosis (before start of CBCT), 1 month after completion of CBCT and one year after CBCT(Fig. 1). Routine medical follow up was done up to 5 years after CBCT. 67 out of the 73 original study participants gave informed consent for blood withdrawal for DNA isolation and could be evaluated in this new analysis.

### Outcome variables

Primary outcome was presence of MetS according to the definition of the National Cholesterol Education Program Adult Treatment Panel III (NCEP ATP III)^31,32^. Within this definition, male patients have MetS when scoring three or more points on the following five components: abdominal obesity (waist circumference > 102 cm), high triglycerides (≥ 1.7 mmol/L or use of medication for dyslipidemia), low HDL cholesterol (< 1.0 mmol/L or use of medication for dyslipidemia), high fasting plasma glucose (≥ 6.1 mmol/L or use of medication for diabetes), or high blood pressure (≥ 130/85 mmHg or use of medication for hypertension). Leucocyte count was measured with a standard Beckman-Coulter blood cell analyzer and expressed as the number of leucocytes x10⁹/L.

Secondary outcome was the development of MetS within a maximum of five years after treatment, which was scored as a ‘persistent MetS status’ – a variable developed for the purpose of our study. Meaning that if a patient has been diagnosed with MetS at some point after the start of CBCT, this would be noted as an event, without the opportunity to reverse this event later on during the follow-up. This results in three groups:

- Group 0: Patients who never developed the MetS.
- Group 1: Patients that newly developed the MetS at a certain time after treatment.
- Group 2: Patients who already had MetS before the start of treatment.

This outcome was used to investigate patients who newly developed MetS within one year, or within five years (group 1), compared to patients who never developed MetS (group 0), or to patients who already had MetS prior to CBCT (group 2).

### Platinum exposure

To investigate the influence of CBCT on the development of MetS and methylation status, calculated platinum exposure (Pt_auc_) during the first year after start of CBCT was used as a variable. For cisplatin, the Area Under the Curve (AUC) represents the cumulative systemic platinum exposure, considering renal clearance throughout the treatment. In all participants, blood samples were drawn during CBCT and follow-up. Serum platinum levels were quantified using inductively coupled plasma mass spectrometry (Varian 820 MS ICP Mass Spectrometer, Varian SPS 3 Sample Preparation System). MwPharm-software (version 3.83) was used to build a three-.

compartment pharmacokinetic model. Other variables that were included in the model were: date of birth, length, weights, creatinine levels, dates of cisplatin administrations, chemotherapy cycle times and the amount of cisplatin per chemotherapy cycle. Renal function was estimated using the Cockcroft-Gault equation. Exposure (AUC, Area Under the Curve, days *mg/L) was determined for each patient during the first year after start of CBCT.

### Assay choices and design

From the isolated DNA the targeted NextGen Bisulfite Sequencing (tNGBS) was performed at EpigenDX, Hopkinton, MA, USA (Panel ID: NGS167, project ID: A882975). Each regulatory element of a gene identified as top candidates in our previous study (DIP2C, ABCG1, ACOT7, BAG4, CACNA1D, CACNA1S, COLEC12, GRHL1, HLCS, HPS1, INTS6L, MFSD2A, NCAM2, ONECUT2, AC090023, TOM1L2). A comprehensive list of these elements, with their ID identifiers, location in human genome, and annotations is presented in Supplementary Table 3.

From the original list of top CpGs, from our previous Norwegian studies, sorted by significance, we prioritized 16 CpGs for further targeted assays based on their potential functionality given a location of CpG in a regulatory region. For this, we have used information if a given CpG was annotated to CpG island, N-shores or N-shelf; 2) if CpG was in close approximation to TSS (defined by distance +- 10 000 bp from CpG); 3. is the CpG part of the binding motive; 4) is there any other evidence from human regulatory databases (i.e. Fantom project); 5. Finally, is the given CpG overlapping with any other human regulatory features (i.e. enhancer, open chromatin, CTCF binding site, enhancer). Semiquantitative score based on presence of the evidence for given CpG in human genome (higher score indicate evidence) gave the final lists. Only specific CpGs from EPIC probes were used excluding probes that might be not specific (see below).

Each element was evaluated before beginning the process of assay design according to following criteria. Gene sequences containing the target of interest were acquired from the Ensembl genome browser and annotated. The target sequences were re-evaluated against the UCSC genome browser for repeat sequences including LINE, SINE, and LTR elements. Sequences containing repetitive elements, low sequence complexity, high thymidine content, and high CpG density were excluded from the in silico design process. Long Interspersed Element (LINE-1), a class of non-Long Terminal Repeat was used to assess changes in global methylation. Pyrosequencing assay that provided an average methylation level of the 36-bp fragment to quantify methylation level of four CpGs was used for quantification. The assay was also performed by EpigenDX.

### Bisulfite modification and multiplexing

Extracted DNA samples (500ng) were bisulfite modified using the EZ-96 DNA MethylationDirect Kit™ (ZymoResearch; Irvine, CA; cat# D5023) per the manufacturer’s protocol with minor modification. The bisulfite modified DNA samples were eluted using M-elution buffer in 46µL. All bisulfite modified DNA samples were amplified using separate multiplex or simplex PCRs. PCRs included 0.5 units of HotStarTaq (Qiagen; Hilden, Germany; cat# 203205), 0.2µM primers, and 3µL of bisulfite-treated DNA in a 20µL reaction. All PCR products were verified using the Qiagen QIAxcel Advanced System (v1.0.6). Prior to library preparation, PCR products from the same sample were pooled and then purified using the QIAquick PCR Purification Kit columns or plates (cat# 28106 or 28183). PCR cycling conditions were as follows: 95 °C 15 min; 45 × (95 °C 30s; Ta°C 30 s; 68 °C 30 s); 68 °C 5 min at 4 °C.

### Library preparation and sequencing

Libraries were prepared using a custom Library Preparation method created by EpigenDx. Constructed library molecules were purified using Agencourt AMPure XP beads (Beckman Coulter; Brea, CA; cat# A63882). Barcoded samples were then pooled in an equimolar fashion before template preparation and enrichment were performed on the Ion Chef™ system using Ion 520™ & Ion 530™ ExT Chef reagents (Thermo Fisher; Waltham, MA; cat# A30670). Following this, enriched, template-positive library molecules were sequenced on the Ion S5™ sequencer using an Ion 530™ sequencing chip (cat# A27764). Any leftovers of libraries and original DNA were destroyed.

### Global methylation

In addition to prioritized CpGs, we included LINE-1 assay that is a marker for global methylation in humans. To assess the general change of methylation level we used a standard pyrosequencing procedure using LINE-1. LINE-1 (Long Interspersed Nuclear Element-1) is commonly used as a marker for global DNA methylation. LINE-1 elements constitute a significant portion of the human genome (~ 17%) and are often used to estimate global DNA methylation levels due to their widespread distribution throughout the genome. Methylation of LINE-1 is typically analyzed because changes in LINE-1 methylation are thought to reflect broader changes in the methylation status of the genome, especially in repetitive elements^33^. This standard assay was also performed by EpigenDx.

### Data analysis

FASTQ files from the Ion Torrent S5 server were aligned to a local reference database using the open-source Bismark Bisulfite Read Mapper program (v0.12.2) with the Bowtie2 alignment algorithm (v2.2.3)^34^. Methylation levels were calculated in Bismark by dividing the number of methylated reads by the total number of reads. Methylation data were encrypted and transferred to secure servers for downstream analysis.

### Imputation of missing variables, and statistical analysis

All analyses were performed in R (v4.0.1), Python (v3.10.0), and SPSS statistics 23.0 (IBM-SPSS, Chicago, IL, USA). Missing variables from patients were imputed using the ‘mice’ package^35^. In case of a missing value for waist circumference, a point for waist circumference was still scored when patients had a BMI ≥ 30 kg/m2, to be able to calculate the MetS in more patients. MetS prior to CBCT had to be imputed for 4 out of 67 individuals and MetS 5 years after CBCT had to be imputed for 4 out of 67 other individuals. In addition to collected previous variables, we have created an additional variable called ‘persistent MetS status’ that describes whether patients developed the MetS at any time point within a maximum of five years after treatment with CBCT. This is also addressed in the paragraph *Outcome variables.*

For the statistical analyses, first the methylation value disparities were assessed at three distinct time points: prior to CBCT, 1 month after completion of CBCT, and 1 year after start of CBCT, utilizing Wilcoxon Signed Rank test for comparison of paired data.

When investigating differences between groups with or without the MetS, Mann-Whitney U test was performed. In case of investigating the differences between three groups, Kruskal Wallis test was used. The significant level for performed tests used was *p* ≤ 0.05.

Subsequently, statistical models were constructed to evaluate the influence of known covariates on the observed data disparities. Primary age and leukocyte levels were utilized as the key covariates in our analysis. We refined our models by excluding patients who already had MetS at the outset. In a sensitivity analysis, we both incorporated and omitted this group to assess the resilience of our predictions. Additionally, we performed sensitivity analyses with both imputed and non-imputed data to validate the robustness of our findings.

By utilizing the variations in methylation levels before CBCT, 1 month after CBCT, and 1 year after CBCT, we developed predictive models. These models considered individual CpGs, combinations of CpGs, as well as the incorporation of BMI and age as a continuous variable to forecast the development of MetS five years after treatment. This analysis serves as a surrogate for assessing the additional clinical predictive value. For predictive purposes, we employed a suite of diverse machine learning models, which included Linear Regression, Random Forest, and Gradient Boosting Classifier from the Scikit-Learn Python package with a 5-fold cross-validation approach applied to this dataset.

We expanded our analysis by stratifying the data, examining how methylation changes vary between patients subjected to high versus low CBCT during treatment (using the mean value as a cutoff). Our objective was to assess the distinct effects of CBCT on patients who had pre-existing MetS compared to those who did not. To achieve this, we utilized generalized linear models that included age and change in leucocyte count as covariates.

## Supplementary Information

Below is the link to the electronic supplementary material.

Supplementary Material 1

## Data Availability

The targeted assays were performed by commercial actor EpigenDX, Hopkinton, MA, USA (Panel ID: NGS167, project ID: A882975). Methylation data were delivered as a percentage of methylation at specific CpG sites targeted by the assay and deposited in the csv format. The datasets generated for this article are not readily available because of the principles and conditions set out in articles 6 (1) I and 9 (2) (j) of the General Data Protection Regulation (GDPR), Dutch and Norwegian Law. However, requests to access the datasets should be directed to the corresponding author and will be facilitated after complying with Ethical Permission and Norwegian Law.

## Abbreviations

ABCG1: ATP Binding Cassette Subfamily G Member 1 - cg06500161
ACOT7: Acyl-CoA Thioesterase 7 - cg08889373
BAG4: BAG Cochaperone 4 - cg14972510
BEP: Bleomycin, etoposide, and platinum
BMI: Body mass index
CACNA1D: Calcium Voltage-Gated Channel Subunit Alpha1 D - cg26408927
CACNA1S: Calcium Voltage-Gated Channel Subunit Alpha1 S - cg04046944
CBCT: cisplatin-based chemotherapy
COLEC12: Collectin Subfamily Member 12 - cg05489343
CpG: Cytosine nucleotide followed by a guanine nucleotide
DIP2C: Disco Interacting Protein 2 Homolog C - cg26561082
GRHL1: Grainyhead Like Transcription Factor 1 - cg14792781
HLCS: Holocarboxylase Synthetase
HPS1: HPS1 Biogenesis Of Lysosomal Organelles Complex 3 Subunit 1 - cg24869056
INTS6L: Integrator Complex Subunit 6 Like
LINE-1: Long Interspersed Element 1
LTR: Long Terminal Repeat Element
MetS: metabolic syndrome
MFSD2A: MFSD2 Lysolipid Transporter A, Lysophospholipid - cg04156896
NCAM2: Neural Cell Adhesion Molecule 2 - cg03877706
ONECUT2: One Cut Homeobox 2 - cg20063141
Pt_AUC_: Platinum exposure
SINE: Short Interspersed Nuclear Element
TC: Testicular cancer
TOM1L2: Target Of Myb1 Like 2 Membrane Trafficking Protein - cg00303773
tNGBS: Targeted NextGen Bisulfite Sequencing
