# Supplementary material for: Changes in methylation associated with development of metabolic syndrome in testicular cancer patients treated with cisplatin chemotherapy
Source: Sci Rep. 2025 Nov 11;15:39452. doi: 10.1038/s41598-025-22918-y (PMC12606202; doi:10.1038/s41598-025-22918-y)
Supplement: Supplementary file 1 — Supplementary Material 1 [file 41598_2025_22918_MOESM1_ESM.docx]

**Supplementary Online Materials for:**

**Changes in methylation associated with development of metabolic syndrome in testicular cancer patients treated with cisplatin chemotherapy**

Marcin W. Wojewodzic*^1,2^, Ellen L.D. Volders*^3^, Coby Meijer^3^, Tom Grotmol^1^, Daan J. Touw^4^, Sjoukje Lubberts^3^, Trine B. Haugen^5^, Jourik A. Gietema^#3^, Trine B. Rounge^#1,6^

^1^Department of Research, Cancer Registry of Norway, Norwegian Institute of Public Health, Oslo, Norway; ^2^Division of Environment and Health, Department of Chemical Toxicology, Norwegian Institute of Public Health, Oslo, Norway; Department of ^3^Medical Oncology and ^4^Pharmaceutics and Pharmacology, University Medical Centre Groningen, University of Groningen, Groningen, The Netherlands; ^5^Department of Life Sciences and Health, Faculty of Health Sciences, Oslo Metropolitan University, Oslo, Norway, ^6^Department of Pharmacy, Faculty of Mathematics and Natural Sciences, University of Oslo, Oslo, Norway.

**Supplementary Table 1:** Basic characteristics of the study population at different time points.

|  | **Prior to CBCT** | | **One month after CBCT** | | **One year after start of CBCT** | | **Five years after start of CBCT** | |
| --- | --- | --- | --- | --- | --- | --- | --- | --- |
|  | **Median/N**  **(25th-75th percentile/%)** | **N** | **Median/N**  **(25th-75th percentile/%)** | **N** | **Median/N**  **(25th-75th percentile/%)** | **N** | **Median/N**  **(25th-75th percentile/%)** | **N** |
| **Age (years)** | 30 (26-36) | 67 | - | - | - | - | - | - |
| **Histology**  **Seminoma**  **Non-seminoma** | 13 (19.4)  54 (80.6) | 67 | - | - | - | - | - | - |
| **Disease stage (Royal Marsden Classification)**  **II**  **III**  **IV** | 54 (80.6)  6 (9.0)  7 (10.4) | 67 | - | - | - | - | - | - |
| **IGCCCG prognosis group**  **Good**  **Intermediate**  **Poor** | 58 (86.6)  8 (11.9)  1 (1.5) | 67 | - | - | - | - | - | - |
| **BMI (kg/m2)** | 25.2 (22.9 - 29.1) | 67 | 25.8 (23.4 - 30.2) | 67 | 25.8 (24.1 - 29.1) | 67 | 25.7 (24.2 - 28.4) | 67 |
| **Persistent MetS status**  **MetS+ prior to CBCT**  **MetS+ newly developed**  **MetS-** | 18 (26.9)  0 (0.0)  49 (73.1) | 67 | - | - | 18 (26.9)  10 (14.9)  39 (58.2) | 67 | 18 (26.9)  15 (22.4)  34 (50.7) | 67 |
| **Leucocyte count x 10^9^/L** | 6.2 (5.3 - 7.3) | 67 | 4.9 (4.1 - 6.4) | 67 | 5.2 (4.2 - 6.6) | 67 | - | - |
| **Platinum exposure**  **(days * mg/L)** | - | - | 58.8 (55.6 - 70.2) | 64 | 67.8 (64.6 - 79.3) | 64 | - | - |

Abbreviations used: CBCT = cisplatin-based chemotherapy; IGCCCG = international germ cell cancer collaborative group; MetS = metabolic syndrome.

**Supplementary Table 2:** Overview of median methylation (%) of all measured CpGs prior to CBCT, one month after CBCT, and one year after start of CBCT, with 25^th^ – 75^th^ percentile and successful measurements (N).* Wilcoxon Signed Ranks Test.

|  | **Prior to CBCT ^A^** | | **One month after CBCT ^B^** | | **One year after start of CBCT ^C^** | | **A vs. B** | | **A vs. C** | |
| --- | --- | --- | --- | --- | --- | --- | --- | --- | --- | --- |
|  | **Median (25th - 75th percentile)** | **N** | **Median (25th - 75th percentile)** | **N** | **Median (25th - 75th percentile)** | **N** | **p^*^** | **N** | **p^*^** | **N** |
| **LINE-1** | 73.7 (72.0 – 75.2) | 66 | 73.9 (72.1 – 74.9) | 65 | 73.9 (72.5 – 75.1) | 64 | .691 | 64 | .162 | 63 |
| **ABCG1** | 67.1 (63.7 – 68.9) | 65 | 68.2 (64.7 – 71.8) | 66 | 67.1 (62.6 – 70.3) | 63 | .051 | 64 | .883 | 61 |
| **ACOT7** | .3 (.0 – .6) | 65 | .3 (.0 – .7) | 64 | .3 (.0 – .7) | 61 | .829 | 62 | .405 | 59 |
| **BAG4** | 48.1 (45.5 – 53.0) | 65 | 51.9 (48.3 – 55.1) | 66 | 48.8 (46.1 – 51.9) | 64 | <.001 | 64 | .530 | 62 |
| **CACNA1D** | 78.6 (76.3- 81.0) | 65 | 76.1 (72.0 – 78.6) | 66 | 75.0 (68.1 – 78.3) | 63 | <.001 | 64 | <.001 | 61 |
| **CACNA1S** | 83.0 (81.0 – 84.1) | 65 | 81.6 (80.0 – 83.9) | 66 | 83.0 (80.8 – 84.6) | 63 | .084 | 64 | .491 | 61 |
| **COLEC12** | 92.4 (91.6 – 93.8) | 65 | 92.7 (91.4 – 93.8) | 66 | 92.8 (91.8 – 93.4) | 63 | .178 | 64 | .889 | 61 |
| **DIP2C** | 95.2 (94.6 – 95.7) | 65 | 95.5 (94.8 – 96.0) | 66 | 95.2 (94.6 – 95.9) | 63 | .284 | 64 | .468 | 61 |
| **GRHL1** | 43.3 (40.1 – 47.8) | 65 | 26.9 (19.9 – 32.3) | 66 | 36.0 (31.7 – 40.1) | 63 | <.001 | 64 | <.001 | 61 |
| **HLCS** | 35.4 (31.8 – 38.9) | 65 | 34.4 (31.9 – 39.0) | 66 | 34.0 (31.2 – 38.7) | 63 | .300 | 64 | .048 | 61 |
| **HPS1** | 93.4 (92.7 – 94.2) | 65 | 93.7 (92.6 – 94.3) | 66 | 937 (93.0 – 94.8) | 63 | .492 | 64 | .052 | 61 |
| **INTS6L** | .3 (.0 – .7) | 65 | .0 (.0 – .5) | 65 | .4 (.0 – .8) | 61 | .016 | 63 | .218 | 59 |
| **MFSD2A** | 93.2 (92.5 – 93.7) | 65 | 92.8 (91.7 – 93.7) | 66 | 92.9 (92.2 – 93.6) | 63 | .077 | 64 | .369 | 61 |
| **NCAM2** | 89.2 (87.3 – 91.1) | 65 | 86.0 (80.2 – 88.2) | 66 | 86.6 (80.4 – 89.6) | 63 | <.001 | 64 | <.001 | 61 |
| **ONECUT2** | 2.5 (2.1 – 3.0) | 65 | 2.3 (2.1 – 3.0) | 66 | 2.5 (1.8 – 3.1) | 63 | .866 | 64 | .980 | 61 |
| **AC090023** | 16.2 (12.5 – 20.2) | 66 | 3.4 (2.5 – 5.4) | 66 | 6.3 (4.6 – 8.9) | 63 | <.001 | 65 | <.001 | 62 |
| **TOM1L2** | 12.7 (9.8 – 15.4) | 66 | 8.0 (6.2 – 10.0) | 66 | 9.4 (7.1 – 11.4) | 63 | <.001 | 65 | <.001 | 62 |


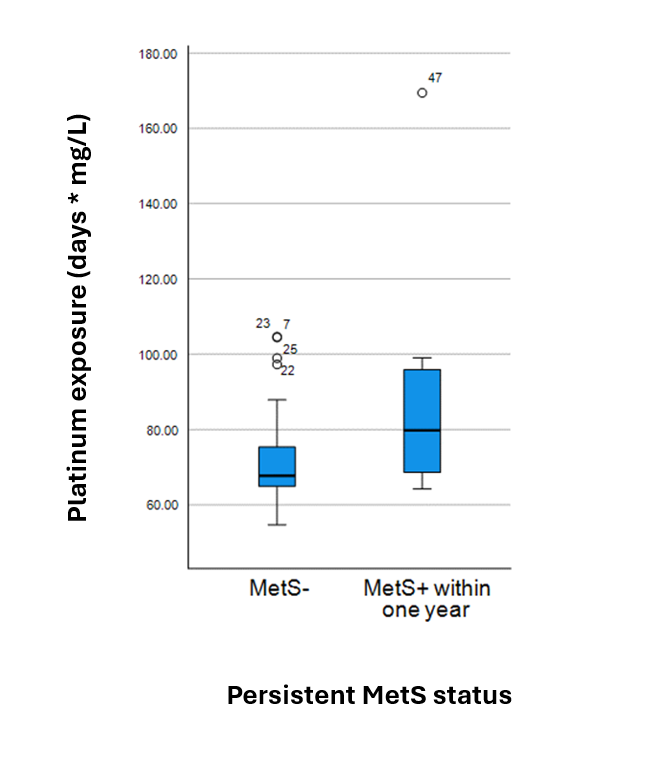


**Supplementary Figure 1:** Platinum exposure (AUC) one year after CBCT in patients without MetS and patients who developed MetS within one year after the start of CBCT, according to the persistent MetS status variable. CBCT = cisplatin-based chemotherapy; MetS = metabolic syndrome


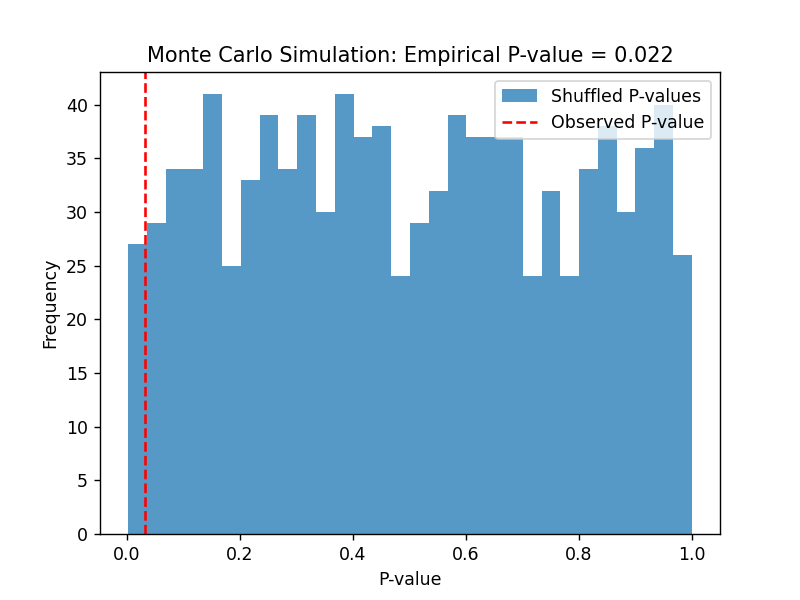


**Supplementary Figure 2:** Distribution of p-values with randomly shuffled labels between groups. This Monte Carlo Simulation assesses the statistical significance of group differences in methylation levels. The original data is used to calculate the OLS (ordinary least squares) regression p-value, which tests whether the group variable significantly explains variation in methylation levels. The groups labels were randomly shuffled 1000 times, and for each reshuffled dataset, a new p-value was calculated. The proportion of randomly shuffled p-values that are smaller than the observed p-value is computed as the empirical p-value, which shows the likelihood that the observed relationship could be due to chance. This analysis shows that our analysis is robust.

**Supplementary Table 3:** Overview of all measured CpGs with their respective information. For prioritization of the top hits from the Norway cohort (DOI: 10.1186/s13148-019-0764-4) we used information if the CpG was annotated to CpG island, N-shores, N-shelf. 2) was close to TSS (defined by distance +- 10 000 bp from CpG); 3. Is the CpG part of the binding motive, 4) were there any other annotation from human regulatory databases (i.e. Fantom project); 5. Was part of any other human regulatory features (i.e. enhancer, open chromatin, CTCF binding site, enhancer). 6. For which targeted assays could be designed. Semiquantitative relative importance score based on presence of the evidence for given CpG in human genome (higher score indicate evidence). Larger weight was assigned to the top CpGs that were located within CpG islands and near transcription start sites (TSS), based on known regulatory biology.

|  | **Position in genome (GCRh38)** | **CpG name** | **Evidence** | **Annotation** | **Relative importance score** |
| --- | --- | --- | --- | --- | --- |
| LINE-1 | distributed evenly | - | literature study | marker of global methylation | 1 |
| ABCG1 | chr21:42236477 | cg06500161 | central CpG from MetS model | lipid metabolism, dyslipidemia, type 2 diabetes | 1 |
| ACOT7 | chr1:6394050 | cg088893373 | island, promoter, transcription factor binding site | Acyl coenzyme family, metabolism, fatty acyl-CoA biosynthesis | 3 |
| BAG4 | chr8:38184637 | cg14972510 | open chromatin | anti apoptotic, negative regulates death | 1 |
| CACNA1D | chr3:53725696 | cg26408927 | enhancer | calcium channels | 1 |
| CACNA1S | chr1:201111502 | cg04046944 | n-shelf, close to TSS, open chromatin | calcium voltage-gated channel, muscles | 3 |
| COLEC12 | chr18:469017 | cg05489343 | central CpGs from MetS model | scavenger receptor collectin subfamily member 12, plays roles in immune responses | 1 |
| DIP2C | chr10:364424 | cg26561082 | s-shore | cellular structure and signal transduction, transcrition regulation | 1 |
| GRHL1 | chr2:9976115 | cg14792781 | expression | metabolism, regulation of metabolism | 1 |
| HLCS | chr21:36967113 | cg23109507 | shore, close to TSS, promoter region | halocarboxylase synthetase, binding of biotin | 3 |
| HPS1 | chr10:98445802 | cg24869056 | n-shore, close to TSS | lysosomal function and the biogenesis of organelles | 2 |
| INTS6L | chrX:135520466 | cg27545041 | central CpG from MetS model | various cellular processes, including gene expression and the integration of RNA polymerase II activity | - |
| MFSD2A | chr1:39954121 | cg04156896 | n-shore, close to TSS | major facilitator superfamily domain containing 2A | 2 |
| NCAM2 | chr21:21200313 | cg03877706 | enhancer, promotor flanking region | neural development and synaptic function | 2 |
| ONECUT2 | chr18:57434733 | cg20063141 | island, close to TSS | cancer related | 2 |
| AC090023 | chr12:65657148 | cg07677157 | central CpG from MetS model, long non-coding RNA | cellular processes, including gene regulation and chromatin remodeling | - |
| TOM1L2 | chr17:17849262 | cg00303773 | close to TSS, binding motive USF1, transcription factor binding site | cellular trafficking, target of myb1-like2 | 4 |
